# The Solid Phase Extraction of Some Metal Ions Using Palladium Nanoparticles Attached to Silica Gel Chemically Bonded by Silica-Bonded N-Propylmorpholine as New Sorbent prior to Their Determination by Flame Atomic Absorption Spectroscopy

**DOI:** 10.1100/2012/764195

**Published:** 2012-05-01

**Authors:** M. Ghaedi, M. Rezakhani, S. Khodadoust, K. Niknam, M. Soylak

**Affiliations:** ^1^Chemistry Department, Yasouj University, Yasouj 75918-74831, Iran; ^2^Chemistry Department, Islamic Azad University, Omidiyeh Branch, Omidiyeh, Iran; ^3^Chemistry Department Faculty of Sciences, Persian Gulf University, Bushehr 75169, Iran; ^4^Chemistry Department, University of Erciyes, 38039 Kayseri, Turkey; ^5^King Saud University, Riyadh, Saudi Arabia

## Abstract

In this research at first palladium nanoparticle attached to a new chemically bonded silica gel has been synthesized and has been characterized with different techniques such as X-ray diffraction (XRD), fourier transform infrared (FT-IR), transmission electron microscopy (TEM), and scanning electron microscopy (SEM). Then, this new sorbent (chemically modified silica gel with N-propylmorpholine (PNP-SBNPM)) was efficiently used for preconcentration of some metal ions in various food samples. The influence of effective variables including mass of sorbent, flow rate, pH of sample solutions and condition of eluent such as volume, type and concentration on the recoveries of understudy metal ions were investigated. Following the optimization of variables, the interfering effects of some foreign ions on the preconcentration and determination of the investigated metal ions described. At optimum values of variables, all investigated metal ions were efficiently recovered with efficiency more than 95%, relative standard deviation (RSD) between 2.4 and 2.8, and detection limit in the range of 1.4–2.7 ng mL^−1^. The present method is simple and rapidly applicable for the determination of the understudied metal ions (ng mL^−1^) in different natural food samples.

## 1. Introduction

The determination and evaluation of trace metals in the various environmental samples is very important and continuously carried out to designate and evaluate their level [1–3]. Direct evaluation of the trace metal content in the complex matrices [3–7] with low concentrations (near or below the instrumentation detection limit) generally has low accuracy. The obviation of these problems simply achieved carrying out an effective extraction and preconcentration procedure prior to measurement step. The solid-phase extraction (SPE) approach as rapid acceptance separation procedure is superior to classical liquid extraction technique in term of rapidity, simplicity, amenability to automation, low disposal costs and lower extraction time [8, 9], and high preconcentration factor.

Inorganic solids surface-functionalized with various organic chelating groups are suitable for adsorption and preconcentration of metal ions [10–12]. SPE procedure charestristic performance significantly can be improved by efficient selection of a suitable sorbent. Silica gel immobilized with various organic compounds was widely used in this technique due its good mechanical and thermal stability, lower swelling susceptibility, shrinking, and microbial and radiation decay [10]. Silica gel generally contain high amount of internal siloxan groups (Si–O–Si) and silanol groups (Si–OH) that its active hydrogen atom is suitable for reaction with organosilyl containing groups to give some organic nature to the precursor inorganic support [13]. 

Nowadays nanometer materials (within the colloidal range and exhibiting typical colloidal properties) due to their special physical and chemical properties including high surface area, high thermal, and chemical stability are very important, and their incorporation to the surface of support significantly improves the SPE performance. Simultaneous chemical modification of supports and attaching metallic nanoparticle to its surface improve the performance of SPE procedure [14, 15].

Therefore, a new sorbent by attachment of palladium nanoparticle to silica gel chemically modified with morpholine was synthesized and characterized. Then, this new sorbent efficiently has been applied for enrichment of trace metal ions. Following optimization, the new sorbent shows great affinity and high adsorption capacities for understudy metal ions, especially in various food samples. The present sorbent and SPE is superior to most previously reported technique due to simple synthesis organic reagent, low consumption of chemical reagents and organic solvent, low cost, good extraction efficiency, lower toxicity, low detection limits and good standard deviations.

## 2. Experimental

### 2.1. Reagent and Material

Asids and bases with highest purity purchased from Merck (Dermasdat, Germany) and used as received. Doubly distilled deionized water, used throughout the experience. Nitrate salts of cadmium (Cd), cobalt (Co), iron (Fe), nickel (Ni), zinc (Zn), magnesium (Mg), barium (Ba), sodium (Na), and potassium (K) were of the highest purity available (Merck, Dermasdat, Germany) and used without any further purification. All the plastic and glassware were cleaned by soaking in dilute HNO_3_ (1 : 9) and were rinsed with distilled water prior to use. The pH adjustment was done by addition of dilute nitric acid or sodium hydroxide to prepare the desired pH solution.

### 2.2. Instruments

**Figure 1:**
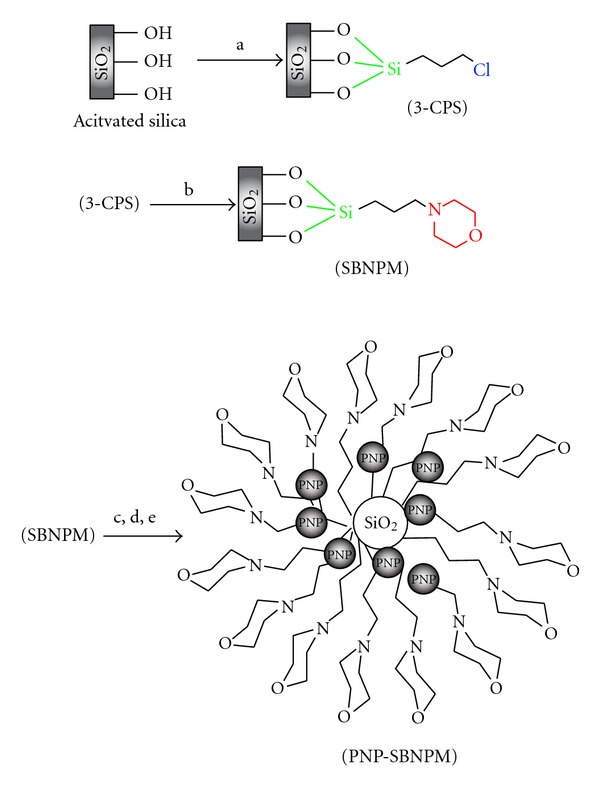
Preparation of PNP-SBNPM catalyst. (a) (3-chloropropyl) trimethoxysilane, toluene, reflux, 18 h. (b) Morpholine, CHCl_3_, Et_3_N, 8 h. (c) Pd(OAC)_2_, ethanol, r.t, 12 h. (d) Washing with ethanol. (e) Washing with ether.

The measurements of metal ions were performed with a Perkin-Elmer AA Analyst 300 (Shelton CT, USA) with hollow cathode lamps and a deuterium background corrector at recommended wavelengths using an air-acetylene flame. The instrumental parameters were those recommended by the manufacturer. A Metrohm 691 pH/Ion meter with a combined glass-calomel electrode was used for adjustment of test solution pH. ^1^HNMR spectra were recorded using Bruker 250 and 500 MHZ. Avance DRX in pure deuterated DMSO-d6 and CDCl_3_ solvents with tetramethylsilane (TMS) as internal standards. Mass spectra recorded on a FINNIGAN-MAT 8430 mass spectrometer operating at 70 eV. X-ray diffraction (XRD, D8, Advance, Bruker, axs) and FT-IR spectroscopy (Shimadzu FT-IR 8300 spectrophotometer) were employed for characterization of the PNP-SBNPM sorbent. Melting points were determined in open capillary tubes in a Barnstead Electrothermal 9100 BZ circulating oil melting point apparatus. TLC accomplished the reaction monitoring on silica gel PolyGram SILG/UV254 plates. Column chromatography was carried out on columns of silica gel 60 (70–230 mesh). Pd nanoparticles on silica-bonded N-propyl morpholine (PNP-SBNPM) were synthesized according to the literature [16] and illustrated in Figure 1.

### 2.3. Preparation of Immobilized Palladium Nanoparticles on Silica-Functionalized Morpholine

#### 2.3.1. Silica-Bonded N-Propyl Morpholine

To a magnetically stirred mixture of 3-chloropropylsilica (5 g) in dry CHCl_3_ (20 mL), morpholine (0.157 g, 1.8 mmol) and some drops of triethyl amine were added and refluxed for 24 h. Then, the mixture was filtered and washed with dichloromethane (3 × 10 mL) and ethanol (3 × 10 mL). After drying in oven, silica-bonded N-propyl morpholine (SBNPM) was obtained as white powder (5.15 g).

#### 2.3.2. Pd Nanoparticles on Silica-Bonded N-Propyl Morpholine (PNP-SBNPM)

To a mixture of silica-bonded N-propyl morpholine (SBNPM) (1 g) in ethanol (10 mL), palladium acetate (0.15 g, 0.67 mmol) was added and stirred 24 h at room temperature. Then, the mixture was filtered and washed with ethanol (3 × 10 mL). After drying in vacuum oven Pd nanoparticles loaded on silica bonded, N-propyl morpholine (PNP-SBNPM) was obtained as dark solid (1.1 g) (Figure 1).

### 2.4. Attachment of Palladium Nanoparticle to the Silica Gel Functionalized with N-Propyl Morpholine

**Figure 2:**
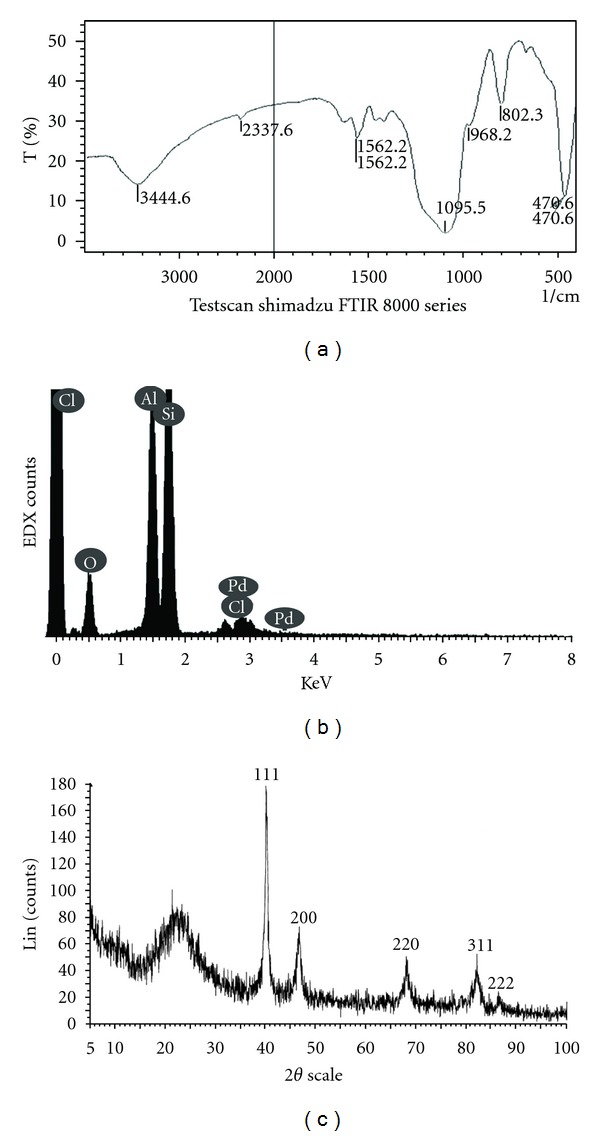
(a) FT-IR Pd nanoparticles on silica-bonded N-propyl morpholine, (b) energy dispersive X-ray spectra (EDX) of PdNP-SBNPM, (c) XRD of PNP-SBNPM.

The palladium nanoparticles silica-bonded N-propyl morpholine (PNP-SBNPM) was produced via the reaction of silica-bonded N-propyl morpholine (SBNPM) with palladium acetate in ethanol [16–22]. For preparation of SBNPM, 3-chloropropylsilica (3-CPS) treatment with morpholine and 3-CPS was produced by the reaction of activated silica with 3-chloropropyl trimethoxysilane (Figure 1). The proposed sorbent also was studied by Fourier FT-IR (Figure 2(a)) as a powerful functional group recognition. As it can be seen, appearance of new peaks around 470.6 (1 cm^−1^), 470.6 (1 cm^−1^), 802.3 (1 cm^−1^), 968.2 (1 cm^−1^), 1562.2 (1 cm^−1^), 2337.6 (1 cm^−1^), 3444.6 (1 cm^−1^) that belong to various functional group of morpholine or its linkage to silica gel efficiently shows the chemical binding of this new reagent to the surface of silica gel.

### 2.5. Column Preparation and Preconcentration Procedure

A short glass column with an inner diameter of 0.5 cm and a length of 50 cm, equipped with porous frits was filled up to a height of about 0.3 cm with a suspension of 0.08 g of proposed sorbent. Palladium nanoparticles silica-bonded N-propyl morpholine- (PdNP-SBNPM) bonded silica gel preconditioned by the blank solution prior to application, and the column was rinsed with water and stored for application.

### 2.6. Preconcentration Procedure

The pH of the solution (250–2000 mL) was adjusted to 7.0 (by addition of dilute HNO_3_ or NaOH) and passed through the column compromise of 0.08 g of this new sorbent at flow rate of 4 mL min^−1^. The adsorbed analytes eluted with 8 mL of 5 M HCl, and their metal ions content were measured by flame atomic absorption spectroscopy (FAAS).

## 3. Results and Discussion

### 3.1. Characterization of Proposed Sorbent

**Figure 3:**
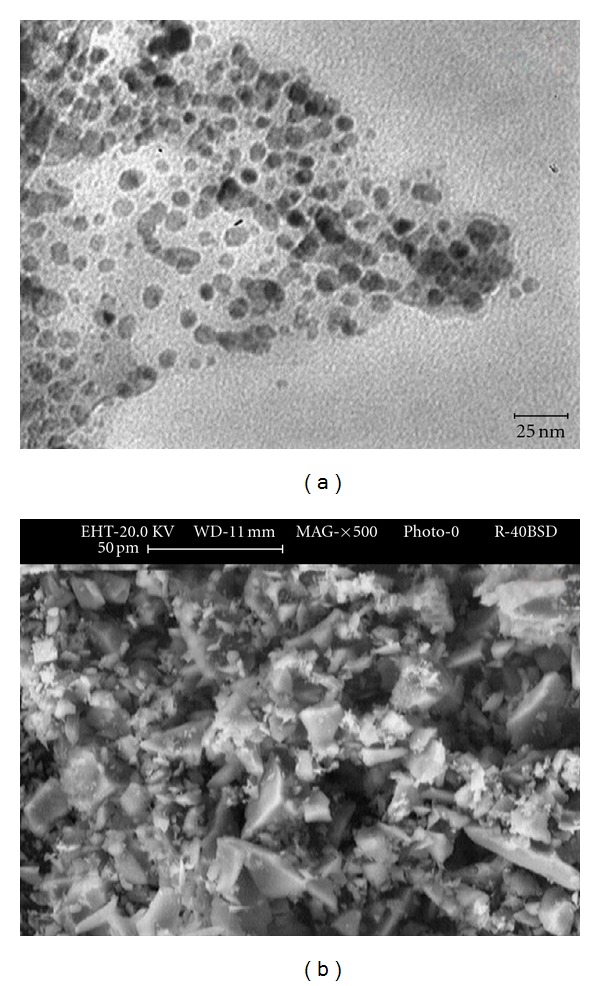
(a) TEM, which shows the image of Pd nanoparticle, (b) SEM of PNP-SBNPM (magnification of 500).

**Figure 4:**
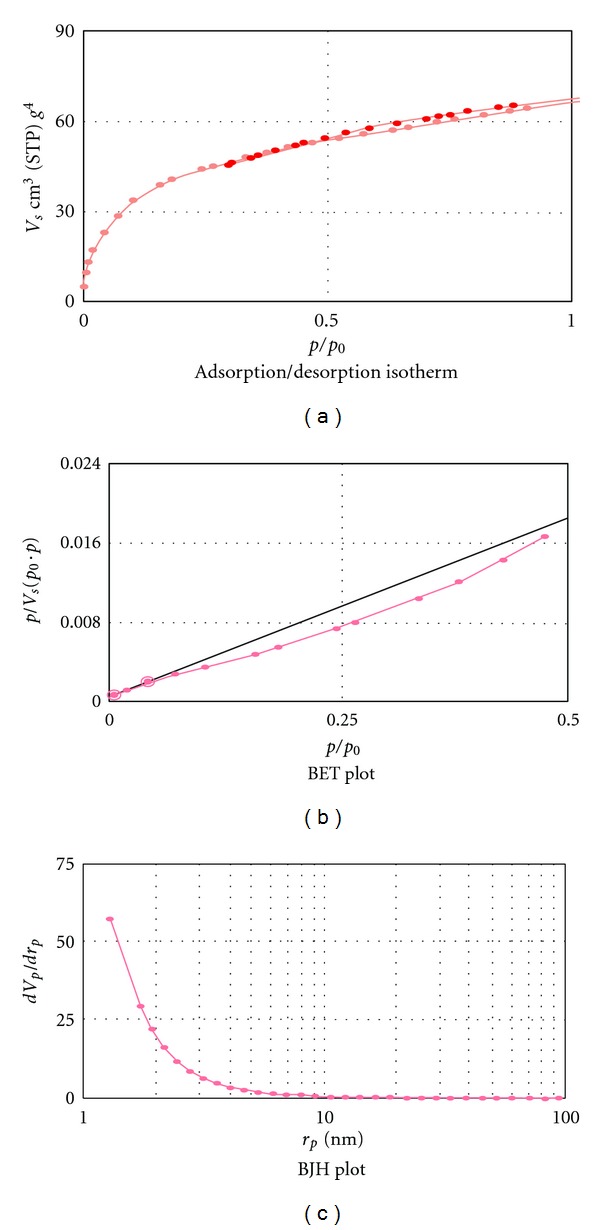
(a) Nitrogen Adsorption-Desorption isotherm for PNP-SBNPM, (b) BET-plot of PNP-SBNPM, (c) BJH average pore diameter diagram for PNP-SBNPM.

The EDX spectrum (Figure 2(b) and Table 1) shows 0.0516 g of palladium for 1 g of proposed sorbent. The XRD pattern of the PNP-SBNPM catalyst (Figure 2(c)) also shows presence of palladium nanoparticle on silica surface. The strongest peaks of the XRD pattern correspond to the SiO_2_, and other peaks are indexed as the (111), (200), (220), (311), and (222) planes belonging to palladium nanoparticle. Transmission electron microscopy (TEM) image of PNP-SBNPM as a new sorbent (Figure 3(a)) shows that the Pd nanoparticles with near spherical morphology are assembled onto silica bonded N-propyl morpholine support with a relatively good monodispersity (±0.8) and an average size of 7 nm. The microscopic features of the catalyst were observed with scanning electron microscopy (SEM) (Figure 3(b)) which efficiently shows the morphology of the silica substrate. A BET surface area of 120 m^2^g^−1^ and a total pore volume of 0.11 cm^3^g^−1^ were measured for this new sorbent.  Figure 4 shows the pore size distribution curve of adsorbents based on the nitrogen equilibrium adsorption isotherm at 77 K, which shows the fine homogenous structure of this new sorbent and efficient immobilization of morpholine and attachment of palladium to the support surface. Table 1 showed the elemental composition of the sample and attachment of palladium nanoparticle to support surface. The support material in SPE should be thermally and chemically stable during the reaction process, and its active sites must be well dispersed on its surface and be easily accessible. Thus, the chemical modification and simultaneous attachment of palladium nanoparticle seems to be efficient pathway for producing new sorbent.

### 3.2. Influences of pH

It is well known that the affinity and tendency of each new sorbent for efficient and selective binding of certain metal ion depend on the nature of its function groups [23–25]. In trace metal enrichment based on chelation, pH has significant influence on the complexation ability and binding fashion of understudy metal ions to the sorbent surface. This is partly because competition of hydrogen ions with metal ions for binding to ligands at low pH and possible formation of low-solubility hydroxide precipitate of metal ions at high pH.

**Figure 5:**
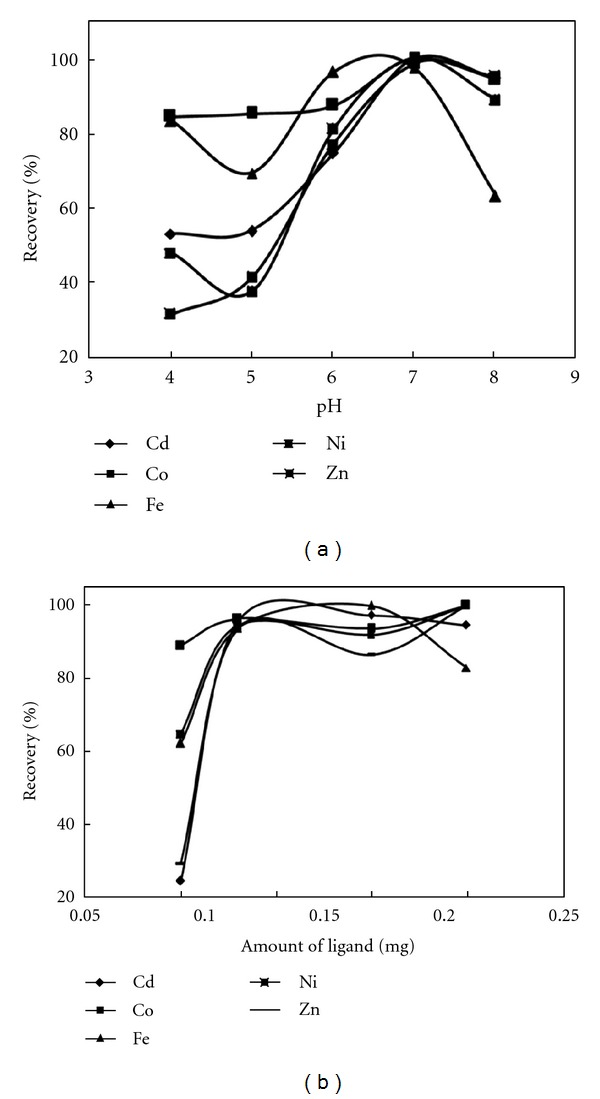
(a) Effect of pH on recoveries of analyte ions (*N* = 3), (b) effect of amount of ligand on recoveries (*N* = 3).

The effect of pH on the sorption and recoveries of understudy metal ions in the pH range of 4.0–8.0 (triplicate) was investigated to determine the precision of the method. At pH > 8.0, the silica gel is prone to hydrolysis. As it is obvious from presented result in Figure 5(a), the new sorbent can quantitatively retain understudy metal ions in the pH of 7.0. The desorption of these understudy retained metal ions by distilled water is very low that show the predominant nature of chelation as a powerful mechanism for trapping and preconcentration of these ions. At pH higher than 7.0, the solution tended to cause the precipitation of M(OH)_n_ or M(OH)^+^ which resulted in the heterogeneity of the fluidic flow and the contamination of sorbents surface and hence a slight decline of the retention efficiency.

### 3.3. Effect of Amount of the New Sorbent on Metal Ion Recoveries

The silica gel itself retained only <30% of these metal ions, the chemically bonded silica gel retained more than 80% while with the palladium nanoparticle-attached chemically modified silica gel retained metal ions more than 95%. Therefore, this new sorbent is capable for quantitative retention and efficient elution of understudy metal ions. The silica gel has profound effects on increasing solid-phase lifetime, reducing the consumption of material and improvement of enrichment factor. 

Therefore, this new sorbent efficiently has been used for trace metal enrichment, and its content was optimized.

The influence of amount of this new sorbent in the range of 0.05–0.25 g has been investigated (Figure 5(b)), and it was seen that increasing amount of sorbent till 0.08 g lead to significant improvement in the recoveries of understudy metal ions. At further value of sorbent at fixed value of all variable especially eluting solution (8 mL of 5 mol L^−1^ HCl) probably due to insufficiency of eluting solution, the recoveries significantly decreased.

### 3.4. Eluent Types and Eluent Volume

The selection of suitable eluent in SPE procedure is very important, and it must be optimized. A suitable and efficient eluent must be able for simultaneous elution of analytes ions with small volume to obtain a high enrichment factor, while does not influence the lifetime and reusability of solid phase. A view glance to the experimental results presented in Table 2 shows that the metal ions sorption was negligible at pH < 5, where one can notice that acidic solutions are the best eluent for reversible elution of understudy metal ions. For this reason, solution of 4.0 mol L^−1^of different acids solutions including HCl, H_2_SO_4_, HClO_4_, and HNO_3_ were applied. It was found that quantitative recoveries for all metal ions were obtained only with 5 mol L^−1^ of HCl (Table 2). The acid concentration was an important factor because at lower proton concentration it may not be enough for quantitative elution of retained metal ions. On the other hand, at high acid concentration determination step may be affected by contamination from applied acid. Generally, efficient elution of analytes from high sample volume by small amount of eluent allows achieving high preconcentration factor. This was examined by changing the volume of 5 mol L^−1^ HCl in the range of 5–12 mL. It was seen that quantitative recoveries could be obtained using 8.0 mL of 5.0 mol L^−1^ HCl.

### 3.5. Effect of Sample Volume

Most of the real samples with complex matrices have low concentrations of trace metals in real samples. Hence, evaluating the maximum enrichable sample volume is necessary. For this purpose, fixed amount of understudy metal ions (50 *μ*g) dissolved in various sample volume in the range of 250–2000 mL, and the retained metal ions was eluted by 8.0 mL of 5.0 mol L^−1^of HCl. It was found that quantitative recoveries for all metal ions were obtained till 1500 mL which was selected as breakthrough volume. By dividing this volume to the final volume solution (8 mL) a preconcentration factor of 187.5 was achieved for all ions.

### 3.6. Adsorption Capacities

The adsorption capacity (most important characteristic performance of every proposed sorbent) shows the required amount of any adsorbent for quantitative trace metal enrichment. Therefore, 0.08 of this new sorbent was equilibrated with 10 mL of 100 *μ*g mL^−1^ of understudy metal ions for 1 h till saturation of all reactive sites of sorbent. The maximum adsorption capacity based on difference between initial and final concentration of all mentioned metal ions was higher than 35 mg g^−1^.

### 3.7. Matrix Effects

The possible interference of other metal ions on the recovery of the analytes metal ions were investigated. A relative error of less than 5% was considered to be within the range of experimental error. The experiments were performed in different levels of the interfering ion concentrations under the optimum conditions of each variable. At the presented results in Table 3 not all of the interfering ions interfered. The recoveries were not less than those in the absence of analyte at all three levels of interfering ion concentrations. These results indicate that this new solid phase has high selectivity towards the mentioned metal ions, and this procedure is applicable for real sample analysis that contains diverse ions level.

### 3.8. Analytical Features

Under the selected conditions, ten portions of standard solutions of all metal ions were enriched and analyzed simultaneously following the general procedure. It was observed that the response of extracted species is linear in the wide concentration range. The experimental preconcentration factors (the ratio of the slope of the calibration graph with and without preconcentration) were 28.3, 22.1, 18.6, 21.9, and 29.3 for Cd^2+^, Co^2+^, Fe^3+^, Ni^2+^, and Zn^2+^ while the preconcentration factor (ratio of volume of initial solution to the eluent volume for all ions were 187.5). In accordance with the definition of IUPAC, the detection limit of the method was calculated based on three times the standard deviation of eight runs of the blank solution, and their values are presented in Table 4.

### 3.9. Application to Food Samples

The feasibility of the methodology has given in Section 2.5 using preconcentration with coated on silica gel for the determination of understudy metal ions in different food samples including sour orange, olive, tangerine, pear, carrot, and strawberry which were treated according to describe procedure in our previous publications [22–24]. Reliability and accuracy of method was assess by treating the real sample according to described procedure in combination with standard addition method, and the results of this study are presented in Table 5 for mention real samples. The satisfactorily reasonable recovery of spiked samples is which gain by standard addition method shows the applicability of method for trace metal enrichment. A good agreement was obtained between the added and measured analyte amounts. The recovery values calculated for the added standards were always higher than 95%, thus confirming the accuracy of the procedure and its independence from the matrix effects. The application of this modified silica gel to preconcentration of sour orange, olive, tangerine, pear, carrot, and strawberry gave high accuracy and precision (%RSD ≤ 5).

## 4. Conclusions

Functionalized silica gel has attracted widespread attention as highly sensitive adsorbent for enrichment of metal ions in the presence of other metal ions. In this study, a unique ligand was chemically bonded to the silica gel and used for preconcentration and separation of Cd^2+^, Co^2+^, Fe^3+^, Ni^2+^ and Zn^2+^, ions as solid-phase extractant. The preparation of ligand-functionalized silica gel was relatively simple and convenient. The proposed sorbent show high affinity, selectivity, and good accessibility for Cd^2+^, Co^2+^, Fe^3+^, Ni^2+^, and Zn^2+^ions. This study also indicated that the design of treeing ligands to silica gel was very suitable for further development of solid-phase extraction techniques.

## Figures and Tables

**Table 1:** EDX property and mass weight of proposed adsorbent.

Element	Weight %	Atomic %	Compd %	Formula
O K	42.07	49.59	57.87	O_2_C
Si K	34.93	23.45	0.00	—
Cl K	1.88	1.00	2.04	Cl_4_C
Pd L	5.16	0.92	0.00	—
C	15.95	25.04	—	—

Totals	100.00	—	—	—

**Table 2:** Effect of type and concentration of eluting agent on recovery of analytes (*N* = 3).

Eluent conditions	Recovery (%)				
	Cd	Co	Fe	Ni	Zn
H_2_SO_4_	57.3	100.0	63.1	51.7	44.1
HNO_3_	12.2	58.2	63.1	49.6	61.8
HCl	67.2	88.0	73.6	80.2	69.8
HClO_4_	58.3	70.5	100.3	42.4	25.6
HCl 3 M	71.2	85.6	75.1	96.4	63.1
HCl 4 M	82.5	88.6	79.1	88.6	70.6
HCl 5 M	94.5	99.5	102.3	94.5	95.6
HCl 7 M	81.3	83.1	89.6	82.4	66.4
HCl 8 M	80.4	72.5	87.4	70.5	59.1
5 mL HCl 5 M	19.8	40.4	52.6	57.8	56.4
7 mL HCl 5 M	54.0	59.8	64.3	70.5	78.1
8 mL HCl 5 M	97.8	102.0	97.6	103.2	98.6
9 mL HCL 5 M	37.1	69.6	75.6	57.8	87.5
12 mL HCl 5 M	22.9	79.7	89.5	68.4	86.6

**Table 3:** Effects of the interferences ions on the recoveries of the examined metal ions.

Foreign ions	Recovery (%)				
	Cd	Co	Fe	Ni	Zn
Mg^+2^	83.7	100	39	73	100
Ba^+2^	72.7	95.5	68	76.7	96.3
Bi^+^	100	71	86.1	100	88.9
Na^+^	72.4	65.5	94.2	44.6	87.1
K^+^	90	69.4	100	32.8	91.3

**Table 4:** Specification of presented method at optimum conditions for each element.

Parameters	Cd	Co	Fe	Ni	Zn
Linear range (ng mL^−1^)	0.01–0.24	0.02–0.34	0.03–0.41	0.015–0.35	0.01–0.26
Detection Limit (ng Ml^−1^)	1.5	2.3	2.7	2.4	1.4
Enrichment factor	28.3	22.1	18.6	21.9	29.3

Preconcentration factor	187.5				

RSD %	2.4	2.6	2.7	2.8	2.7
Recovery %	97.1	95.6	96.8	97.4	97.3

**Table 5:** Recovery studies of trace metal ions from some real sample.

Real samples	Ions	Added (*μ*g/g)	Found (*μ*g/g)	Recovery %	RSD %
Sour orange	Ni	0.0	0.94		3.9
		0.5	1.47	106.0	3.1
	Cd	0.0	0.10		4.1
		0.5	0.62	104.0	3.5
	Zn	0.0	10.65		3.0
		0.5	11.12	94.0	2.6
	Fe	0.0	15.52		3.2
		0.5	16.01	98.0	2.1
	Co	0.0	0.19		3.8
		0.5	0.70	102.0	3.4

Olive	Ni	0.0	1.08		3.1
		0.5	1.60	104.0	2.6
	Cd	0.0	0.13		4.2
		0.5	0.64	102.0	3.8
	Zn	0.0	11.13		3.7
		0.5	11.66	106.0	3.0
	Fe	0.0	14.92		3.8
		0.5	15.40	96.0	3.4
	Co	0.0	0.20		3.7
		0.5	0.69	98.0	3.1

Tangerine	Ni	0.0	0.30		3.9
		0.5	0.81	102.0	3.6
	Cd	0.0	0.15		4.3
		0.5	0.68	106.0	3.8
	Zn	0.0	10.47		2.9
		0.5	11.00	106.0	2.5
	Fe	0.0	13.73		3.1
		0.5	14.21	98.0	2.5
	Co	0.0	0.21		3.9
		0.5	0.70	97.0	3.4

Pear	Ni	0.0	0.23		3.8
		0.5	0.72	98.0	3.4
	Cd	0.0	0.11		3.5
		0.5	0.62	102.0	3.0
	Zn	0.0	12.73		3.0
		0.5	13.25	104.0	2.6
	Fe	0.0	14.51		3.0
		0.5	15.02	102.0	2.4
	Co	0.0	0.34		3.7
		0.5	0.83	98.0	3.2

Carrot	Ni	0.0	0.22		3.8
		1.0	1.21	99.0	3.2
	Cd	0.0	0.40		3.6
		1.0	1.42	102.0	3.3
	Zn	0.0	15.11		3.9
		1.0	16.13	102.3	3.4
	Fe	0.0	17.15		3.1
		1.0	18.10	95.4	2.8
	Co	0.0	0.41		3.8
		1.0	1.45	104.0	3.5

Strawberry	Ni	0.0	0.20		4.2
		1.0	1.18	98.0	3.6
	Cd	0.0	0.25		3.8
		1.0	1.21	96.0	3.3
	Zn	0.0	13.15		3.2
		1.0	14.10	95.0	2.6
	Fe	0.0	14.95		3.0
		1.0	15.99	104.0	2.6
	Co	0.0	0.31		3.8
		1.0	1.36	105.0	3.3
